# 

**Published:** 1811-05

**Authors:** 


### MONTHLY CATALOGUE OF MEDICAL BOOKS.

A Letter to the Physicians and Surgeons of St. George's Hospital, on Mr. Davy's simple Galvanic Circles, considered as a Topical Assistant Branch of Medicine in the correction of disordered living action; founded upon the Theories of Dr. Garnet and Mr. Davy, and confirmed by practical experience, by Matthew Yatman, esq. 8vo. Callow.

Hortus Kewensis, or a Catalogue of the Plants cultivated in the Royal Botanic Garden at Kew. By the late William Aiton, vol. 2, 8vo. Longman and Co.

The Return to Nature, or difference of the Vegetable Regimen, with some account of an Experiment made during the last three or four years in the Author's family. By John Frank Newton, esq. Part I. 8vo. Cadell.

### NOTICES TO CORRESPONDENTS.

Communications from the following gentlemen were received too late for publication this month :

Charles Batham, M. D. Messrs. W. H. Leadbeater, J. Ring, A. Fogo, G. Nasse Hill, J. Smith, Apothecarius, an Essex Practitioner, S. L. and T.

We have been informed that the Practitioner of Hackney, mentioned in the Monthly Magazine, and alluded to in the account of Stramonium in this Number of our Journal, as having the merit of introducing the practice of smoking that plant in cases of asthma, is Mr. Toulmin.

The notice of Mr. Dumbell's Liverpool Lint, of which we have heard a good character, was mislaid, and did not arrive in time for insertion.

*Printed by E. Hemsted, Great New Street, Fetter Lane.*

